# MIRTFnet: Analysis of miRNA Regulated Transcription Factors

**DOI:** 10.1371/journal.pone.0022519

**Published:** 2011-08-17

**Authors:** Haroon Naeem, Robert Küffner, Ralf Zimmer

**Affiliations:** Institut für Informatik, Ludwig-Maximilians-Universität München, Germany; George Mason University, United States of America

## Abstract

**Background:**

Several expression datasets of miRNA transfection experiments are available to analyze the regulatory mechanisms downstream of miRNA effects. The miRNA induced regulatory effects can be propagated via transcription factors (TFs). We propose the method MIRTFnet to identify miRNA controlled TFs as active regulators if their downstream target genes are differentially expressed.

**Methodology/Principal Findings:**

MIRTFnet enables the determination of active transcription factors (TFs) and is sensitive enough to exploit the small expression changes induced by the activity of miRNAs. For this purpose, different statistical tests were evaluated and compared. Based on the identified TFs, databases, computational predictions and the literature we construct regulatory models downstream of miRNA actions. Transfecting miRNAs are connected to active regulators via a network of miRNA-TF, miRNA-kinase-TF as well as TF-TF relationships. Based on 43 transfection experiments involving 17 cancer relevant miRNAs we show that MIRTFnet detects active regulators reliably.

**Conclusions/Significance:**

The consensus of the individual regulatory models shows that the examined miRNAs induce activity changes in a common core of transcription factors involved in cancer related processes such as proliferation or apoptosis.

## Introduction

Transcription factors (TFs) and microRNAs (miRNAs) are prominent gene regulatory factors [1]. TFs are proteins that bind to promoter of genes to regulation their expression and miRNAs are small (∼22-nucleotides) noncoding RNAs that regulate the mRNA stability and translation through the action of the RNA-induced silencing complex (RISC) [1]–[3]. miRNAs play an important role in several biological processes such as cell development, differentiation and various diseases including cancer [4]–[5].

Several databases have been developed to improve the research of miRNAs based on their target genes [6]–[8]. TarBase [9] and miRecords [10] collect target genes of the miRNAs in different organisms. miRSel [11] provides putative miRNA-gene associations extracted from biomedical abstracts by text mining. Several computational algorithms have been developed to computationally predict target genes/sites of miRNAs, such as PITA [12], PicTar [13], TargetScan [14] and miRanda [15].

Patterns of gene silencing induced by miRNA are achieved by mRNA degradation or translational inhibition [16]. Several transcription profiling studies of miRNA transfection experiments have been conducted to investigate the influence of miRNAs on transcript levels [17]–[19]. These experiments show that miRNAs exert a widespread impact on the regulation of their target genes and (potentially mediated via TFs) on non-target genes. TFs have been found enriched among miRNA targets in plants [20] and insects [21].

This paper aims at the determination of the TFs active in a miRNA induced expression measurements. This can be difficult as they are frequently regulated on the protein level (e.g. by phosphorylation) that is not immediately detectable by transcriptional profiling. On the other hand, transcriptional effects of miRNAs are in general expected to be small and could easily be obscured by noise in the measurements. The detection of active TFs thus requires very sensitive approaches that rely on indirect evidence rather than the expression of the regulators themselves. Sohler *et al.* [22], Essaghir *et al.* [23] and Liu *et al.* [24] independently proposed the hypergeometric test to detect active TFs. According to our analyses, the hypergeometric (HG) test is not sensitive enough to pick up the small expression changes caused by miRNAs. Analogously, statistical tests such as the HG test were applied to detect expression changes of miRNAs based on the expression of their target genes [25]–[31].

In contrast, Tu *et al*. [25] suggested linear models to detect miRNA regulated TFs based on the differential expression of their target genes. From miRNA transfection experiments, they extracted two layered networks where TFs mediate miRNA initiated regulatory effects to explain observed expression changes in miRNA and TF target genes. The time complexity of their approach substantially limited the set of detected TFs. On average, only two active TFs were identified per transfection experiment [25].

We propose a simple method called MIRTFnet targeted at the determination of regulated TFs. Similar to work presented in [22]–[25], our method analyzes the expression patterns of the known TF targets to distinguish active from inactive TFs. In contrast to [25] our approach is targeted at the comprehensive determination of the involved TFs. In contrast to [22]–[24], MIRTFnet is more sensitive and, thus, also suited for detecting the relatively minor effects induced by miRNA activity. After we determine a set of active regulators, we construct network models by connecting regulators and their targets by known miRNA-target, TF-target and kinase-TF relationships. We analyzed 43 individual transfection experiments and discuss differences and overlaps between the resulting regulatory models.

## Materials and Methods

### Datasets

#### Gene expression datasets

We obtained 43 gene expression profiles of 18 different miRNA transfection studies in different human cell lines. Selbach *et al.* [17] measured gene expression data in HeLa cells at 8 h and 32 h after miRNA overexpression of miR-155, miR-16 and let-7b. Expression profiles by He *et al.* [32] include gene expression changes at 24 h after miRNA overexpression of miR-34 family (i.e., miR-34a and miR-34b), in six different cell lines (e.g., HeLa, A549 H1-term and TOV21G H1-term). Georges *et al.* [33] measured p53-inducible miRNAs, miR-192 and miR-215, at 10 h and 24 h after miRNA transfection in a human cell line (i.e., HCT116 Dicer -/- #2). Baek *et al.* [34] measured the gene expression data in HeLa cells at 24 h after miR-124, miR-1 and miR-181a transfection. We also use the dataset by Grimson *et al.* [35] that measured gene expression data in HeLa cells at 12 h and 24 h after miRNA overexpression of miR-7, miR-9, miR-122, miR-128, miR-132, miR-133, miR-142 and miR-181a.

For brevity, results and discussion will focus on 25 experiments [17], [33]–[34] unless noted otherwise. See the supplement for results on the full set of experiments and for additional analyses. All analyses are based on comparing mRNA levels between transfection and control via log_2_ fold-changes (l_2_fc).

#### miRNA-gene associations

Human miRNA-gene associations were obtained from the text mining derived database miRSel [11] and the curated databases TarBase [9], miRecords [10] and miR2Disease [5]. We also obtained putative human miRNA-target pairs predicted by PITA [12], PICTAR [13] and TargetScan [14] (Table 1). The PITA miRNA target predictions were compiled using a more stringent threshold (from –6 to –20) to reduce the number of false positive predictions.

**Table 1:** Associations between regulators and their targets.

Databases	Regulators	Kind	Target genes	Pairs
**MiRSel**	486	miRNA	1969	7604
**TarBase**	110	miRNA	837	1023
**MiRecords**	93	miRNA	614	772
**miR2Disease**	176	miRNA	364	596
**PITA**	640	miRNA	14065	307465
**PICTAR**	163	miRNA	5975	44403
**TargetScan**	249	miRNA	9446	110172
**UCSC**	106	TF	3997	16688
**JASPAR**	66	TF	12261	73878
**TRANSFAC**	219	TF	304	794
**HPRD**	462	Kinase	1800 proteins	4182

#### TF-gene associations

Human TF-gene regulatory relationships were predicted as described in [36] using the position specific weight matrices (PWM) from the JASPAR database. We used relationships from the human genome browser at UCSC (http://genome.ucsc.edu/) [25]. Additionally, we collected TF-gene associations from TRANSFAC [37] (ver. 2005), see Table 1. We refer to these TF-gene relations as JASPAR, UCSC, and TRANSFAC, respectively.

#### Protein-protein interactions

Human protein-protein interactions (PPIs) have been downloaded from the Human Protein Reference Database (http://www.hprd.org/) [38]. Using PPIs, miRNA-gene associations and TF gene relations, we compile the miRNA-kinase associations and kinase-TF including miRNA-kinase-TF physical interaction relationships, see Table 1. We compile all types of interactions into a gene network.

### MIRTFnet: Determining active miRNAs and TFs

TFs might be activated or inhibited by modifications (e.g. phosphorylation) that cannot directly be detected by microarray measurements. Activity changes of miRNAs and TFs can still be determined by analyzing whether the expression levels of their putative downstream targets (according to Table 1) could be a sample from the background distribution of the remaining (i.e. non-target) genes. The probability of this null hypothesis (p-value) can be derived by a number of statistical tests described below. Resulting p-values are multiple testing corrected using the Benjamini and Hochberg method [39]. For corrected p-values of less than 0.05 the null hypothesis is rejected for the respective miRNAs and TFs. We refer to such regulators as active regulators in the tested experiment. Both miRNAs and TFs can be tested given lists of experimentally validated or computationally predicted targets.

TFs are also assumed to be active if they exhibit a fold change of at least two or less than 0.5 in a given expression experiment. Active miRNAs cannot be identified this way as they have not been measured on the arrays.

#### Wilcoxon test

We apply the Wilcoxon nonparametric rank-sum (WR) method [27]–[28], [40] to test the null hypothesis that the regulator targets exhibit no significant rank differences in comparison to other genes (non-targets). Ranks were derived by sorting the genes based on their log fold changes between transfected and wild type measurements. If the rank distributions of targets and non-targets are significantly different the null hypothesis will be rejected. Then, targets of the tested regulators exhibit greater log fold changes than non-targets according to the test.

#### Kolmogorov-Smirnov test

Whether or not the distributions of (miRNAs and TFs) target and non-target genes are shifted with respect to each other can also be tested by another non-parametric test, the Kolmogorov-Smirnov (KS) test. Both WR and KS tests do not require the selection of thresholds. Both tests have not yet been applied to TF activity detection, only to predict transfecting miRNAs [25], [27]–[29]. While the KS test should only test for distribution shifts, the WR test is also sensitive to shape differences in the two distributions [41]–[43]. Nevertheless, both tests usually yield consistent results as found by e.g. Gsponer *et al.* [40]. MIRTFnet therefore reports TF activity changes only if they are identified by both tests.

#### Hypergeometric test

We also apply the hypergeometric test to detect active transcription factors as proposed by Sohler *et al.* [22], Essaghir *et al.* [23] and Liu *et al.* [25]. The HG test is used to calculate the significance of the TFs including the transfecting miRNA in a given experiment as p-value of the observed over-representation of TF/transfecting miRNA targets among the differentially expressed genes. The WR, KS and HG test will be applied to the same set of miRNA and TF target genes (Table 1) and used the same procedure for multiple testing correction (Benjamini-Hochberg). To enable the comparison to Essaghir *et al*., we follow their approach to regard genes as regulated if they exhibit a fold change of more than 2 or less than 0.5. Both WR and KS tests do not require such a threshold but exploits the ranks of all genes that have been measured. Note that we apply both tests on the set of TF targets as obtained from databases and predictions (Table 1). In contrast, Essaghir *et al.* augmented curated databases by their own manual literature searches.

### Model of miRNA actions

We construct network models of miRNA downstream actions. Here, we aim to connect the transfecting miRNA to TFs via miRNA-TF, TF-TF and kinase-TF interactions derived from databases and computational predictions (Table 1). Thus, TFs are included if they were active according to WR and KS test as described in the last section and are reachable from the transfecting miRNA by a path of known or predicted interactions. Note that kinases are included as connectors between miRNAs and TFs in the models although the activity of kinases has not been determined in the examined studies. Thereby, we aim to give explanations for expression changes observed after miRNA transfection. Based on these models we evaluate to what extent expression changes could potentially be explained based on the current knowledge of causal interactions.

**Figure 1:**
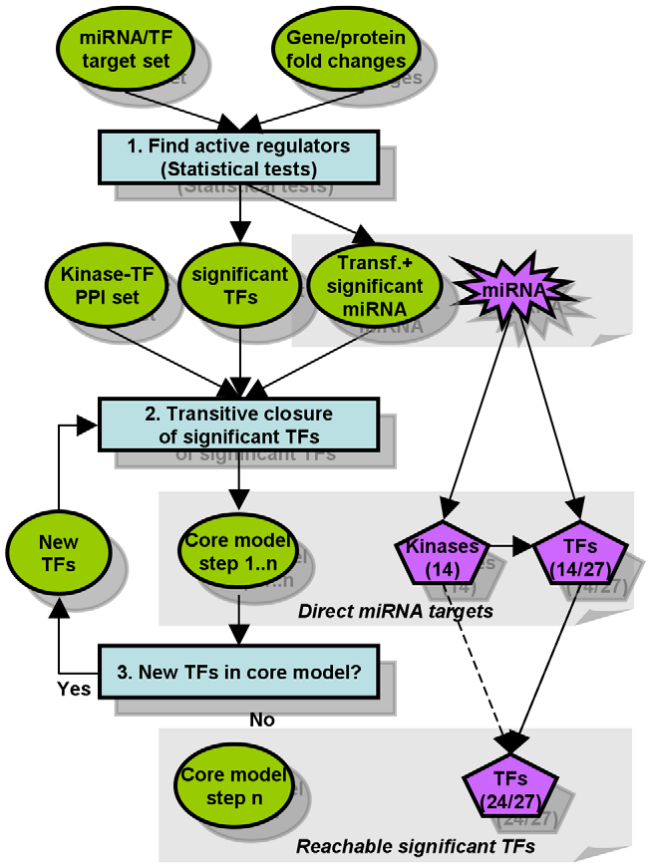
Modelling miRNA actions from expression measurements. Active regulators such as miRNAs and TFs are detected by their effect on the expression of downstream targets, here exemplified by the Wilcoxon test. In step 1 just the direct miRNA targets (kinases and significant TFs) are added to the model. Additional significant TFs are included if they can be connected to the model by interactions from Table 1, i.e. by repeating steps 2 and 3. The model of miR-155 transfection (8 hr), for instance, includes 14 kinases and 24 out of 27 TFs detected as active by MIRTFnet. The remaining 3 TFs could not be connected by known interactions. Using these models we consider gene expression changes observed after miRNA transfection as explained if they satisfy two constrains: (1) such a gene must be targeted by an active TF, and (2) such a TF must be connectable to the transfecting miRNA by a path of known or predicted miRNA-TF, TF-TF and kinase-TF interactions.

Thus, we propose a cascade of TF activation steps (Figure 1) including the transfecting miRNA, kinases and TFs. Genes that are directly and exclusively affected by miRNAs will most likely be inhibited. This is not necessarily true for indirectly affected TFs or TF target genes.

## Results

### Evaluation of the transfecting miRNAs

We first evaluate how well the miRNAs used for transfection (called primary miRNAs) are detected by MIRTFnet. Only for these miRNAs we can be certain that they should be recognized as active. By using miRNA targets from predictions and databases, transfecting miRNAs were recognized in 42 out of 43 miRNA transfection experiments. In most experiments, p-values for transfecting miRNAs were well below the alpha value of 0.05 (Table 2). This suggests that active regulators can be detected reliably by miRTFnet. Here we find that the WR test identifies 98% of the transfecting miRNAs. Only 79% and 42% of the transfecting miRNA were identified by the KS and HG tests, respectively (see Table 2 and File S1). The re-detection of the transfecting miRNA from differential expression of the miRNA targets has also been described in [25]–[31], where similar recall rates have been reported.

**Table 2:** Prediction of active miRNAs based on miRNA targets derived from databases and predictions.

Datasets	Transfecting miRNA	Cell line	Time point	Primary miRNA detected		
				WR Test	KS Test	Hyper. Test
Selbach et al., 2008	miR-155	Hela	8	√	√	-
	miR-155	Hela	32	√	-	√
	miR-16	Hela	8	√	√	-
	miR-16	Hela	32	√	√	√
	let-7b	Hela	8	-	-	-
	let-7b	Hela	32	√	√	-
Georges et al., 2008	miR-192	HCT116	24	√	-	√
	miR-192	HCT116	10	√	-	√
	miR-215	HCT116	10	√	-	-
	miR-215	HCT116	24	√	-	√
Baek et al., 2008	miR-124	Hela	24	√	√	-
	miR-1	Hela	24	√	√	-
	miR-181a	Hela	24	√	√	-
He et al., 2005	miR-34a	A549	24	√	√	-
	miR-34b	A549	24	√	√	-
	miR-34a	HCT116	24	√	√	√
	miR-34b	HCT116	24	√	√	-
	miR-34a	TOV21G	24	√	√	-
	miR-34b	TOV21G	24	√	√	-
	miR-34a	DLD	24	√	√	√
	miR-34b	DLD	24	√	√	-
	miR-34a	HeLa	24	√	√	√
	miR-34b	HeLa	24	√	√	-
	miR-34a	A549 p53	24	√	√	-
	miR-34b	A549 p53	24	√	√	-

The transfecting miRNAs have been detected as active in 24 out of 25 miRNA transfection experiments based on the Wilcoxon (WR) test. 8 out of 10 transfecting miRNAs in 19 out of 25 miRNA transfection experiments have been identified as active applying the Kolmogorov-Smirnov (KS) test. While 6 out of 10 transfecting miRNAs in 9 out of 25 miRNA transfection experiments have been detected applying the hypergeometric test.

In addition to recall, we propose to also analyze the specificity of detection. Therefore, we also analysed how many other miRNAs (called secondary miRNAs) are statistically shown to be active in response to miRNA transfection experiments. We assessed the performance of each method by the area under the receiver operating characteristic (AUROC) curve, a measure combining specificity and recall. Here, we considered primary miRNAs as positive examples and secondary miRNAs as negative examples. This assessment might underestimate the true performance, for instance if miRNA transfection causes activity changes in secondary miRNAs that we count as false positives. Using miRNA-gene target associations from databases and prediction tools, the WR test achieves an AUROC of 0.90, KS AUROC of 0.86 and hypergeometric test AUROC of 0.65. The AUROC is further increased to 0.91 if only those primary miRNAs were considered that are found statistically active by both the WR and KS test.

### Detection of active transcription factors

#### Wilcoxon test

Active TFs (Table 3) were detected: 1) if they exhibit a fold change of at least two in a given miRNA transfection experiment or 2) via the differential expression of their direct downstream targets (obtained from JASPAR, UCSC and TRANSFAC) using statistical tests as described in method section. In the Selbach *et al*. [17] miRNA-transfection datasets for instance, we identified more than 20 active TFs (e.g., ELK4, CREB1, E2F1 and MAFB) and 10 TF based on fold change (e.g., TP53, ZEB1, ZNF423, FOSB and FOX03) applying the Wilcoxon (WR) test. We have also found five TFs (FOS, CREB1, ID1, ZNF423 and MYB) that are both statistically significant and differentially expressed in the miR-155 (32 hr), let-7b (32 hr), miR-34a and miR-34 b (24 hr) miRNA transfection experiments. Overall, 86 TFs have been detected applying the WR test.

**Table 3:** Prediction of active TFs based on the expression of their target genes.

Dataset	Transfec. miRNA	Cell line	Time point(hr)	Total	Fold chng.	FC+ stat. sig.	Statistically (stat.) sig. TFs				
							WRtest	KS test	Shared KS+ WR	HGtest	Shared HG+ WR
Selbach et al., 2008	miR-155	Hela	8	27	7	0	20	6	6	0	0
	miR-155	Hela	32	30	11	3	16	13	13	6	3
	miR-16	Hela	8	20	9	0	11	2	2	1	0
	miR-16	Hela	32	34	10	0	24	17	17	0	0
	let-7b	Hela	8	27	5	1	21	9	9	5	3
	let-7b	Hela	32	25	8	1	16	9	9	5	4
Georges et al., 2008	miR-192	HCT116	24	19	2	0	17	12	12	1	0
	miR-192	HCT116	10	25	0	0	25	12	12	0	0
	miR-215	HCT116	10	20	0	0	20	10	10	0	0
	miR-215	HCT116	24	20	1	0	19	10	10	1	0
Baek et al., 2008	miR-124	Hela	24	33	4	0	29	31	28	0	0
	miR-1	Hela	24	40	2	0	38	34	34	0	0
	miR-181a	Hela	24	22	5	0	17	24	17	0	0
He et al., 2005	miR-34a	A549	24	64	0	0	64	62	61	0	0
	miR-34b	A549	24	57	0	0	57	56	52	0	0
	miR-34a	HCT116	24	65	4	1	60	58	55	0	0
	miR-34b	HCT116	24	66	3	0	63	59	58	0	0
	miR-34a	TOV21G	24	71	0	0	71	59	59	0	0
	miR-34b	TOV21G	24	64	0	0	64	62	62	0	0
	miR-34a	DLD	24	65	1	1	63	60	59	0	0
	miR-34b	DLD	24	59	3	1	55	53	51	0	0
	miR-34a	HeLa	24	64	0	0	64	62	60	0	0
	miR-34b	HeLa	24	63	0	0	63	57	56	0	0
	miR-34a	A549 p53	24	61	0	0	61	60	58	0	0
	miR-34b	A549 p53	24	59	0	0	59	60	56	0	0
**Total**				**111**	**38**	**5**	**86**	**71**	**69**	**11**	**7**

Overall, 86 TFs have been identified by MIRTFnet (applying the Wilcoxon test (WR)) with the used datasets. 71 TFs have been detected applying the Kolmogorov-Smirnov (KS) test and 11 TFs have been detected using the hypergeometric test. The hypergeometric test does not find any significant TF in 19 out of 25 miRNA transfection profiles.

#### Kolmogorov-Smirnov test

In addition to the WR test we also applied the Kolmogorov-Smirnov (KS) test and the hypergeometric test. The KS test identified in total 71 active TFs (Table 3). 69 out of 71 KS active TFs have also been identified by the WR test. In most of the cases, the TFs identified by the KS test are a subset of those identified by the WR test.

#### Hypergeometric test

As proposed by Sohler *et al.* [22], Essaghir *et al.* [23] and Liu *et al.* [24], we also applied the hypergeometric (HG) test (equivalent to Fisher's test). The HG test identified only very few active TFs (11, see Table 3). The HG p-values were consistently higher (less significant) than the p-values derived from the WR and KS test.

### Rank distribution of active TFs

**Figure 2:**
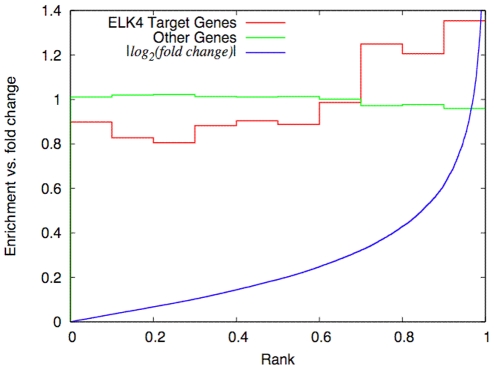
Rank distributions of the 1826 putative ELK4 targets (red) vs. the 16101 remaining genes (green). The ranks are derived from the list of target genes sorted according to their fold changes (blue) in the miR-155 transfection experiment at 32 h. The distributions are normalized to show the relative overrepresentation of ELK4 targets in histogram bins of |*log_2_*(*fold change*)| >0.4 (corresponding to fold changes >1.3, or <0.75) ELK4 targets are enriched by about 50% compared to |*log_2_*(*fold change*)| <0.4. ELK4 is thus identified as an active regulator with a p–value of 2.87E-11 according to the Benjamini-Hochberg corrected the Wilcoxon test.

**Figure 3:**
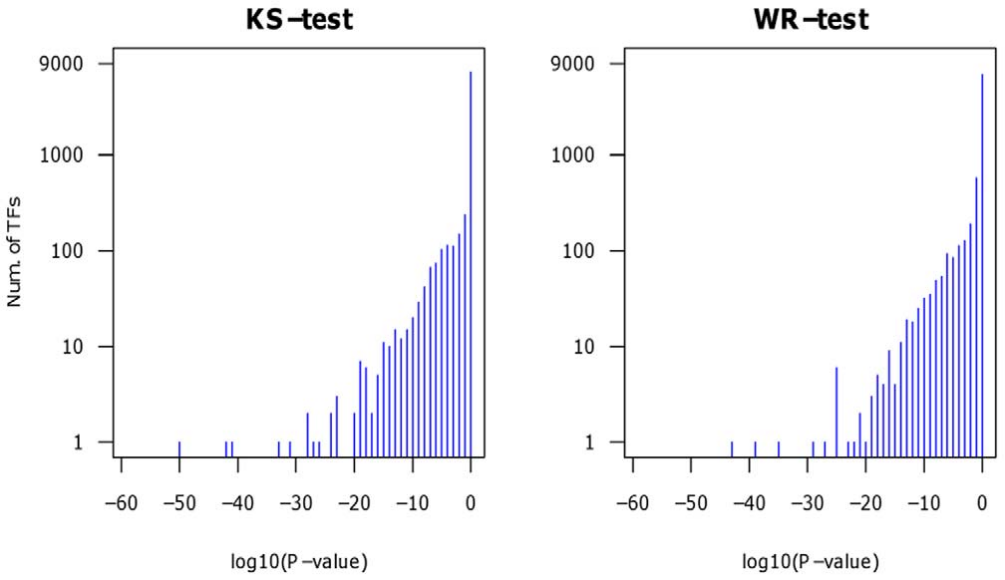
P-value distribution of TFs in miRNA transfection experiments. To detect active TFs, the statistical tests have been applied to 196 TFs across 43 transfection profiles. If a test assigns a p-value of less than 0.05 after multiple testing correction a given TF is identified as active for the given measurement. Depicted are the p-value distributions of the WR and KS tests, i.e. the number of TF that fall in a given p-value range according to the respective test.

We detect regulators such as miRNAs and TFs as active via the expression of their putative target genes. If the mean expression of the target genes is significantly different to the mean expression of the remaining genes we identify the corresponding regulator as active according to the applied tests. As an example for an active transcription factor we depict ELK4 in the transfection experiment of has-miR-155 at 32 h (Figure 2). JASPAR predicts 1826 putative targets of ELK4. Compared to the 16101 remaining genes, ELK4 targets exhibit larger fold changes and thus higher ranks than expected by chance. Interestingly, the enrichment of differentially expressed ELK4 target genes is already noticeable at moderate fold-changes (>1.3, or <0.75). Similar distributions are observed for other TFs as well (not shown). Note that Figure 2 serves only as visualization whereas active TFs are only determined by the statistical tests described above. To summarize the analysis of all TFs across all miRNA transfection profiles, we show the p-value distributions as derived from WR and KS tests in Figure 3.

### Randomized Testing

We also evaluate if TF are detected as active by chance. Here, we randomize the association of gene names and expression levels in each experiment and apply the WR and KS test as described in the methods section. We shuffle gene labels and expression levels randomly 100 times. The test did not find a single regulator as active (neither miRNA nor TF) at a corrected p-value of less than 0.05 after applying multiple testing correction using the method of Benjamini and Hochberg [39]. This was true regardless of which sub-selections of miRNA-target or TF-target data sources were used. For instance, in case of miRNA-targets we tested curated databases, databases plus low recall prediction tools (e.g., PICTAR and TargetScan) and databases plus high recall prediction tools (e.g., PICTAR, TargetScan and PITA).

### Global expression pattern explained by active TFs

Based on protein-protein interactions, miRNA-targets and TF-targets, we constructed transfection experiment specific models that connect the transfecting miRNA via causal relationships to the TFs that were detected as active using the proposed statistical tests.

In each transfection profile, 196 TFs were tested. On average, 23 TFs were detected as active by both WR and KS test. Here, 21 out of 23 TFs could be connected to the transfecting miRNA based on causal relationships (compare File S4).

We used miRNA-targets and TF-targets from curated databases as well as computational predictions (Figure 1).

We analyzed to what extent regulators (e.g., miRNAs and TFs) and their known/predicted target genes can explain the overall expression changes observed on the microarrays. File S1 shows the gene regulation that can be explained by MIRTFnet via miRNA-TF relations. The identified TFs and their target genes thus provide a potential explanation for the majority (on average 67%, File S1 shows the exact numbers for each measurement) of the observed differential expression in the examined miRNA transfection experiments.

### miRNA-target TF associations in databases and prediction programs

**Figure 4:**
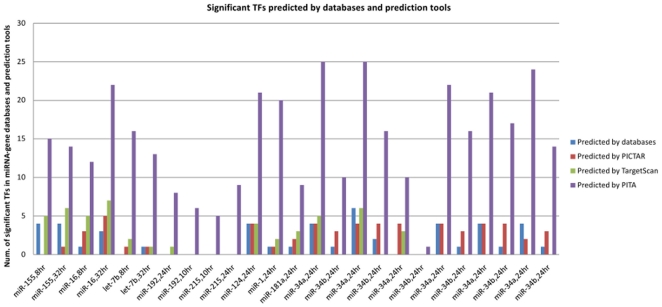
Significant TFs predicted by databases and/or sequence prediction programs of miRNA-target genes. TFs are detected as active by analyzing the expression levels of their downstream targets (the Wilcoxon test). Active TFs can be predicted from databases in 18 out of 25 miRNA transfection experiments (on average 3 TFs per miRNA transfection experiment). PICTAR and TargetScan prediction programs can predict on average 3 and 4 active TFs in 19 and 16 out of 25 miRNA transfection experiments, respectively. PITA can predict TFs in all 25 miRNA transfection experiments (on average 15 active TFs per transfection profiles). To improve recall, the miRNA-gene associations of databases and prediction programs are combined (on average 16 active TFs per transfection).

Whether a connection between the transfecting miRNA and active TFs can be established depends on the current databases and sequence based prediction programs of miRNA target genes (Figure 4).

Based on these associations we aim to construct models of miRNA actions (see methods). However, these would be very small if only databases as well as PICTAR and TargetScan are used for model construction. Here, only four TFs on average would be connected to the transfecting miRNA. To improve this recall, PITA miRNA-gene associations are used as well. The combined miRNA-gene associations suggest connections to about 16 active TFs for all of the examined miRNA transfection experiments.

### Detected TFs and their reported roles in cancer – literature mining

miRNAs play potential roles in the pathogenesis of different diseases including cancer [44]–[45]. Some miRNAs may be directly involved in cancer development by controlling cell differentiation and apoptosis or by targeting cancer oncogenes and/or tumor suppressors [46]–[48]. All of the transfection experiments analyzed in the present paper have been described in the literature as cancer relevant.

The miR-192, miR-215 and miR-34 experiments were conceived because these miRNAs are reportedly regulated by p53 and are thus potentially involved in cancer related processes [32]–[33]. We also analyzed the miR-155, let-7 and miR-16 transfection experiments [17] for which interactions with p53 have been reported as well [49]–[50]. We thus expect to predominantly identify cancer related TFs which we will evaluate below as a proof of concept of MIRTFnet. The cancer specific involvement of many of the TFs MIRTFnet determined as active is indeed discussed in the literature.

In case of the miR-155 transfection, we detected a range of oncogenic TFs (e.g., SPI1, MYCN, MAFB, FOS and REL) and the tumor suppressor TP53, which may suggest a tumor-induction effect. Previous reports have experimentally confirmed that SPI1 (Pu.1) reduces the transcriptional activity of p53 tumor suppressor family [51]. The deregulation of MYCN leads to undergo cell cycle exit and terminal differentiation [52]–[53].

In the miR-16 transfection, we found the target genes of oncogenic TFs (e.g., MAFB, MYB) including Cyclin D1/CCND1 and CDK6 to be differentially expressed as well. Both CCND1 and CDK6 are experimentally validated targets of miR-16 that induce cell cycle arrest [54]–[55].

In case of let-7b transfection, the tumor suppressor TP53 and oncogenes such as E2F1, FOS and FOSB have been found active, which might hint to tumor-suppressing effects of let-7b. Recently, the let-7 family miRNAs were found to inhibit E2F family oncogenes [25]. The TFs (e.g., TP53, FOS and FOSB) are predicted targets of let-7b [12]. The let-7 family is described to be in many human cancers [56]–[57].

Recent studies confirm that TP53 regulates apoptosis by targeting miRNAs, such as miR-34, miR-192 and miR-215 [46], [58]–[60]. The miR-34, miR-192 and miR-215 halt cell cycle progression by co-ordinately targeting transcripts that play critical roles in mediating cell cycle control [33], [61]–[62]. Our results showed that miR-34 alters the activity of the MYCN, MYB, MAFB and E2FI oncogenes, all being involved in apoptosis and cell proliferation [62]–[63]. The predicted target of miR-34, YY1 has been shown to down-regulate TP53 [64]. miR-192 and miR-215 were found to inhibit HOXA10 and several oncogenes (e.g., MYCN and MAFB). Furthermore, miR-192 and miR-215 were found to down-regulate CDC7, which might provide an additional explanation for the involvement on miRNAs in the p53 pathway to mediate cell cycle and apoptosis [65].

Also in case of miR-9 transfection, tumor suppressor p53 and oncogene transcription factors such as Runx1, E2F1, MYCN and MYB have been found active. Both MYC and MYCN oncoproteins act on the mir-9-3 locus and cause activation of miR-9 expression in tumor cells [66]. Runx1 is an experimentally validated target of miR-9 and has been reported to act as tumor suppressor, dominant oncogene or mediator of metastasis [67]-[68]. In case of miR-122, TFs such as MAFB and SRF have been found active. SRF is an experimentally validated target of miR-122 and it regulates cell proliferation, differentiation, and cytoskeletal reorganization [69].

### miRNA-TF regulatory model upstream and downstream of TP53

The literature discussed in the previous section implies the involvement of the examined miRNAs and the identified TFs in cancer related processes. For a proof of concept of MIRTFnet, we analyze whether this common background is also reflected by a common set of TFs active across several of these experiments. Therefore, we compiled individual regulatory models (Figure 1) from all examined miRNA transfection experiments. The detailed models (provided upon request) characterize the miRNA downstream actions in terms of kinases as well as active TFs that are mutually connected by interactions from databases or computational predictions. Interestingly, these models show substantial overlaps. In the following, we discuss the two intersection models constructed from the TFs and/or kinases contained in the regulatory networks (1) upstream and (2) downstream of TP53 that are contained in at least 7 of 19 individual models. By analyzing transfection experiments of sets of functionally related miRNAs we found that each set addresses a common core of transcription factors specific for that set.

**Figure 5:**
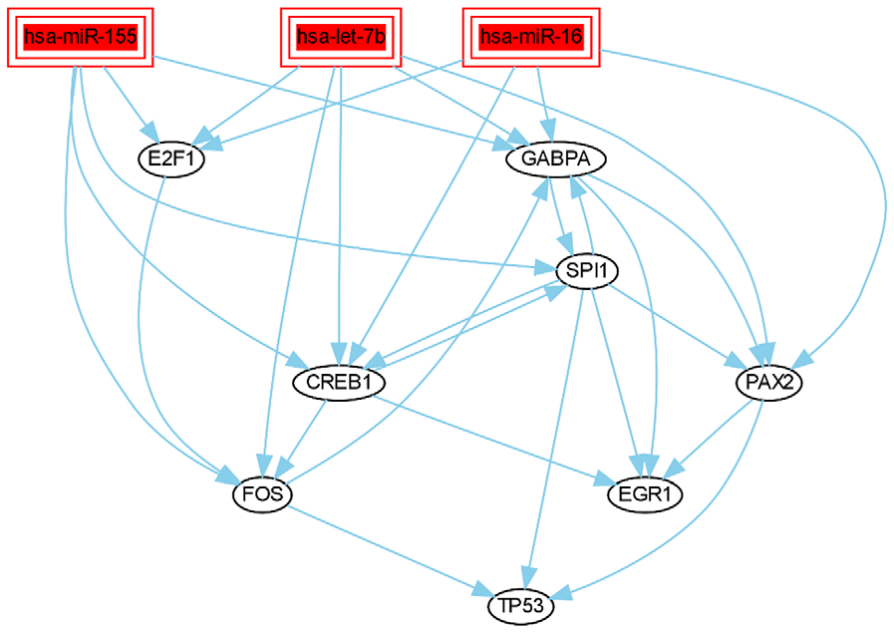
Upstream of TP53: intersection of miR-155, miR-16 and let-7b models. Individual regulatory models of the miR-155, miR-16 and let-7b transfection experiments are compiled by MIRTFnet and intersected. These models show substantial overlaps, regulating directly or indirectly oncogene TFs (such as TP53, FOS, CREB1). The interaction of these miRNAs with p53 have also been reported in the literature [49]–[50].

**Figure 6:**
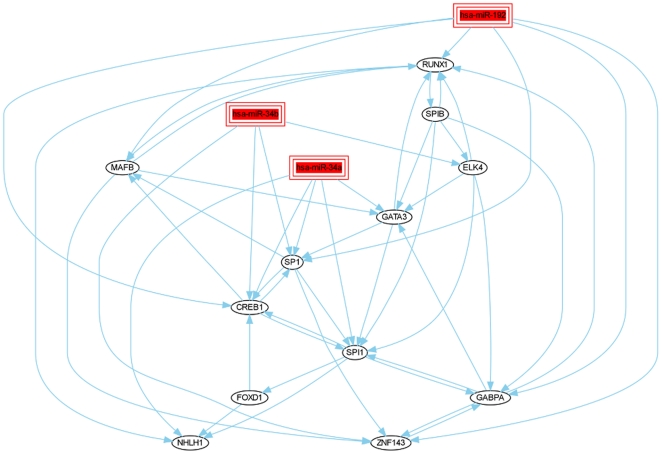
Downstream of TP53: intersection of miR-34a, miR-34b and miR-192 models. As in Figure 5, individual models of miR-192/215 and miR-34 microRNAs have been intersected. The shown microRNAs are reportedly regulated by TP53 and are thus potentially involved in cancer related processes [46,58–60].

The upstream miRNAs such as miR-155, miR-16 and let-7b are found to regulate TP53 [49]-[50]. The miRNAs miR-34, miR-192 and miR-215 are found to be regulated by the p53 transcription factor [32]–[33], [46]. The upstream intersection model including miR-155, miR-16 and let-7b miRNA transfection, shows that these miRNAs regulate the tumor suppressor TP53 and oncogene TFs (e.g., FOS, E2F1) (Figure 5). In comparison to the upstream model, in the downstream intersection model miR-34a/b, miR-192 miRNA transfection were found to regulate oncogeneic TFs (e.g., MAFB, ELK4, GATA3) (Figure 6). A minority of TFs is part of the upstream and downstream miRNA-TF models. These TFs regulate common oncogene TFs (e.g., CREB1, SPI1, etc). Thus, although the detected active TFs are all involved in cancer (further substantiated in the following section), the two regulatory models are quite distinct demonstrating that specific results are obtained from analyzing different sets of miRNAs.

### Pathway and Gene Ontology analyses of the regulatory model

Here, we disregarded 6 examined datasets to avoid a bias towards miR-34. The intersection model contains 21 TFs and 34 kinases. We first analyzed the contained kinases. Kinases were included because of miRNA-kinase-active TF links, i.e. they usually do not receive direct support from the expression measurements. According to a pathway analysis using DAVID [70], these kinases are associated with several KEGG signalling pathways including the MAPK, cancer, cell cycle and apoptosis pathways. 17 out of 34 kinases are part of the KEGG MAPK signalling pathway (e.g. MAPK9, MAPK8, CHUK, NLK and MAPK14). The MAPK signalling pathway is immediately connected to the p53 signaling pathway. 12 kinases are also part of the KEGG cancer signaling pathway (e.g. PTK2, MAPK3 and SKP2).

Notably, most of the active TFs detected by our approach are well known for their involvement in cancer. According to the DAVID analyses in pathway databases such as KEGG or BioCarta, only cancer related pathways were detected with statistical significance. These included the KEGG pathways ‘prostate cancer’, ‘pancreatic cancer’, ‘apoptosis’ and ‘pathways in cancer’, which account for 10 of the TFs identified as active (i.e., ELK4, NFKB1, TP53, FOS, SPI1, CREB1, RELA, REL, E2F1 and ARNT). According to enrichment analysis of GO terms (DAVID), the TFs in the intersection model are associated with 101 GO categories including cell differentiation.

For instance, CREB1 as well as the NFκB TF complex (NFKB1, RELA, REL) trigger cell survival and cell proliferation processes. Four additional TFs are oncogenes (REL, ELK4, MYB and MAFB). Another two TFs (PAX5 and SP1) are involved in cell differentiation, which also is a cancer-associated process. For the remaining TF YY1 associations with cancer through p53 regulation have been reported in the literature [64]. The relationships between 19 of the 21 TFs as derived from the STRING database [71] are depicted in the supplement (File S1).

We provide details on the examined miRNAs, kinases and TFs as well as their interactions (Files S1, S2, S3, S4, S5). In addition to the above definition of a core model, the supplementary material thus enables analyses on arbitrary combinations of the individual models.

## Discussion

Transcription factors (TFs) are important factors regulating gene expression. Based on miRNA transfection experiments, methods have been suggested previously [in 22–25] detecting TFs that mediate at least some of the observed expression changes of genes not directly targeted by the transfecting miRNA. As an alternative, we proposed the method MIRTFnet for the determination of TFs regulated in response to the miRNA transfection. In comparison to the approach presented in [25], our method is more sensitive as all TFs with known targets can be tested with little computational effort. MIRTFnet can detect the small expression changes in the TF target genes caused by the transfection with miRNAs. In contrast to previous methods based on the hypergeometric (HG) test [22]–[24], MIRTFnet does not require the manual filtering of TF targets to enable the detection of regulated TFs.

MIRTFnet identifies active TFs by analyzing the differential expression in the TF target genes. Despite the dependency of our method on TF target gene predictions, the detection of active regulators is very reliable. In the examined experiments, Wilcoxons rank-sum test (WR) detected the transfecting miRNAs more reliably than the Kolmogorov-Smirnov (KS) and hypergeometric (HG) tests (recall: WR = 42/43 = 98%, KS = 34/43 = 79%, HG = 18/43 = 42% and AUC: WR = 90%, KS = 86%, HG = 65%). The AUC improves to 91% if only those TFs are considered active that are detected by both WR and KS. Therefore, MIRTFnet reports TFs as active regulators if they are identified by both tests.

A transfecting (primary) miRNA might also lead to activity changes in secondary miRNAs, which we will analyze and discuss in a forthcoming paper.

The miRNAs used in the transfections examined in this paper were predominantly selected by the authors of the corresponding studies because of their reported involvement in cancer. In case of the detection of active TFs, we thus expected MIRTFnet to predominantly propose cancer related TFs. We could clearly confirm this expectation, and thereby ensure the reliability of our active TF predictions, as the involvement in cancer is indeed known for almost all of our detected TFs.

Starting from the transfecting miRNA, we constructed putative models based on known or predicted regulator (i.e. miRNA, TF and kinase) target relationships. For each examined transfection experiment, most of the detected TFs could be connected directly or indirectly to the transfecting miRNA. Indirect connections in our models included miRNA-kinase-TF and miRNA-TF-TF relationships. Our models provide potential explanations for the majority of the observed expression changes as all known TFs were tested by MIRTFnet. These models also contained relationships to unregulated genes. This is not surprising as many genes might be regulated in a synergistic fashion, i.e. require different regulators being active at the same time. Relationships to unregulated genes might also be caused by incorrect target predictions.

An additional unexpected result stems from intersecting the proposed regulatory models constructed for the individual miRNAs. We detected several active TFs across many different transfection studies. This could potentially suggest common regulatory mechanisms downstream of cancer relevant miRNAs. At the same time, the responses of TFs to different subsets of miRNAs can be quite distinct depending on whether these miRNA act either upstream or downstream of p53 (Figures 5 and 6).

Our results further reinforce the growing awareness that these small non-coding RNAs have an intrinsic function in gene regulatory networks including TFs related to key cellular contexts such as cell proliferation and apoptosis.

## Supporting Information

File S1 **Additional Data information.** The File S1 describes the information contained in additional data files including figures, tables and results extracted from the miRNA transfection experiments analyzed in the present paper using MIRTFnet.
(DOC)

File S2 **miRNA-target associations.** The File S2 contains the transfecting miRNA target genes including TFs, kinases and other differentially regulated miRNA target genes. For more details see File S1.
(CSV)

File S3 **Kinase-TF relationships.** The File S3 lists the kinase-TF associations necessary to link active TF to the transfecting miRNA in each miRNA-transfection experiment. For more details see File S1.
(CSV)

File S4 **Significant TFs.** The File S4 contains the TFs identified as active (either based on the Wilcoxon test or based on the fold change criterion) in each miRNA transfection experiment. For more details see File S1.
(CSV)

File S5 **TF-target relationships.** The identified active TFs and their target genes (in addition to the direct miRNA target genes) provide a potential explanation for the majority of the observed differential expression in the 25 examined miRNA transfection experiments. The File S5 contains the active TF regulated target genes information. For more details see File S1.
(CSV)
