# Supplementary material for: MIRTFnet: Analysis of miRNA Regulated Transcription Factors
Source: PLoS One. 2011 Aug 17;6(8):e22519. doi: 10.1371/journal.pone.0022519 (PMC3157336; doi:10.1371/journal.pone.0022519)
Supplement: File S1 — Additional Data information. The File S1 describes the information contained in additional data files including figures, tables and results extracted from the miRNA transfection experiments analyzed in the present paper using MIRTFnet. (DOC) [file pone.0022519.s001.doc]

# MIRTFnet: Analysis of miRNA regulated transcription factors

## Additional Data information

## miRNA-transfection profiles mapping

We obtained 43 gene expression profiles of 10 different miRNA transfection studies in several human cell lines. Selbach *et al.* [1] measured gene expression data in HeLa cells at 8h and 32h after miRNA overexpression of miR-155, miR-16 and let-7b. Expression profiles by He *et al.* [2] include gene expression changes at 24h after miRNA overexpression of miR-34 family (i.e., miR-34a and miR-34b), in six different cell lines (e.g., HeLa, A549 H1-term and TOV21G H1-term). We also use the data of Georges *et al.* [3] that measured p53-inducible miRNAs, miR-192 and miR-215, at 10h and 24h after miRNA transfection in a human cell line (i.e., HCT116 Dicer -/- #2). A dataset by Baek *et al.* [4] on miR-124, miR-1 and miR-181a (24h after transfection) was also examined. We also use the dataset by Grimson *et al.* [5] that measured gene expression data in HeLa cells at 12h and 24h after miRNA overexpression of miR-7, miR-9, miR-122, miR-128, miR-132, miR-133, miR-142 and miR-181a. Table A1 lists experiment and dataset ID for each miRNA transfection profile. Not all of the datasets were counted for the miRNA-TF core regulatory network (see below) to avoid a bias towards miRNA 34.

## Table A1 - miRNA transfection experiments analyzed in the present paper

| **Datasets** | **Transf. miRNA** | **Cell Line** | **Experiment-ID** | **Dataset- ID** | **Counted** | **Time point** |
| --- | --- | --- | --- | --- | --- | --- |
| **Selbach et al., 2008** | hsa-miR-155 | Hela | pub19053 | pub19053-1 | Yes | 8hr |
|  | hsa-miR-155 | Hela | pub19053 | pub19053-2 | Yes | 32hr |
|  | hsa-miR-16 | Hela | pub19053 | pub19053-3 | Yes | 8hr |
|  | hsa-miR-16 | Hela | pub19053 | pub19053-4 | Yes | 32hr |
|  | hsa-let-7b | Hela | pub19053 | pub19053-5 | Yes | 8hr |
|  | hsa-let-7b | Hela | pub19053 | pub19053-6 | Yes | 32hr |
| **Georges et al., 2008** | hsa-miR-192 | HCT116 Dicer -/- #2 | GSE13105 | GSM328287 | Yes | 24hr |
|  | hsa-miR-192 | HCT116 Dicer -/- #2 | GSE13105 | GSM328290 | Yes | 10hr |
|  | hsa-miR-215 | HCT116 Dicer -/- #2 | GSE13105 | GSM328291 | Yes | 10hr |
|  | hsa-miR-215 | HCT116 Dicer -/- #2 | GSE13105 | GSM328288 | Yes | 24hr |
| **Baek et al., 2008** | hsa-miR-124 | Hela | GSE11968 | GSM302945 | Yes | 24hr |
|  | hsa-miR-1 | Hela | GSE11968 | GSM302946 | Yes | 24hr |
|  | hsa-miR-181a | Hela | GSE11968 | GSM302995 | Yes | 24hr |
| **He et al., 2005** | hsa-miR-34a | A549 H-1 term | GSE7864 | GSM190752 | Yes | 24hr |
|  | hsa-miR-34b | A549 H-1 term | GSE7864 | GSM190753 | Yes | 24hr |
|  | hsa-miR-34a | HCT116 Dicer -/- #2 | GSE7864 | GSM190756 | Yes | 24hr |
|  | hsa-miR-34b | HCT116 Dicer -/- #2 | GSE7864 | GSM190757 | Yes | 24hr |
|  | hsa-miR-34a | TOV21G H1-term | GSE7864 | GSM190760 | No | 24hr |
|  | hsa-miR-34b | TOV21G H1-term | GSE7864 | GSM190761 | No | 24hr |
|  | hsa-miR-34a | DLD Dicer -/- #2 | GSE7864 | GSM190764 | No | 24hr |
|  | hsa-miR-34b | DLD Dicer -/- #2 | GSE7864 | GSM190765 | No | 24hr |
|  | hsa-miR-34a | HeLa | GSE7864 | GSM190768 | No | 24hr |
|  | hsa-miR-34b | HeLa | GSE7864 | GSM190769 | No | 24hr |
|  | hsa-miR-34a | A549 p53-1026 | GSE7864 | GSM190772 | Yes | 24hr |
|  | hsa-miR-34b | A549 p53-1026 | GSE7864 | GSM190773 | Yes | 24hr |
| **Grimson et al., 2007** | miR-7 | Hela | GSE8501 | GSM210896 | No | 12hr |
|  | miR-7 | Hela | GSE8501 | GSM210897 | No | 24hr |
|  | miR-9 | Hela | GSE8501 | GSM210898 | No | 12hr |
|  | miR-9 | Hela | GSE8501 | GSM210899 | No | 24hr |
|  | miR-122 | Hela | GSE8501 | GSM210900 | No | 12hr |
|  | miR-122 | Hela | GSE8501 | GSM210901 | No | 24hr |
|  | miR-128 | Hela | GSE8501 | GSM210902 | No | 12hr |
|  | miR-128 | Hela | GSE8501 | GSM210903 | No | 24hr |
|  | miR-132 | Hela | GSE8501 | GSM21904 | No | 12hr |
|  | miR-132 | Hela | GSE8501 | GSM210905 | No | 24hr |
|  | miR-133a | Hela | GSE8501 | GSM210906 | No | 12hr |
|  | miR-133a | Hela | GSE8501 | GSM210907 | No | 24hr |
|  | miR-142-3p | Hela | GSE8501 | GSM210908 | No | 12hr |
|  | miR-142-3p | Hela | GSE8501 | GSM210909 | No | 24hr |
|  | miR-148b | Hela | GSE8501 | GSM210910 | No | 12hr |
|  | miR-148b | Hela | GSE8501 | GSM210912 | No | 24hr |
|  | miR-181a | Hela | GSE8501 | GSM210913 | No | 12hr |
|  | miR-181a | Hela | GSE8501 | GSM210896 | No | 24hr |

**Table A2 - Prediction of active miRNAs based on miRNA targets derived from databases and predictions, identified by MIRTFnet- Wilcoxon (WR), Kolmogorov-Smirnov (KS) and hypergeometric (HG) test.**

| **Datasets** | **Transfect./ over expressed miRNA** | **Cell line** | **Time point** | **Primary miRNA detected** | | |
| --- | --- | --- | --- | --- | --- | --- |
|  |  |  |  | **WR test** | **KS test** | **HG test** |
| **Selbach et al., 2008** | miR-155 | Hela | 8 | √ | √ | - |
|  | miR-155 | Hela | 32 | √ | - | √ |
|  | miR-16 | Hela | 8 | √ | √ | - |
|  | miR-16 | Hela | 32 | √ | √ | √ |
|  | let-7b | Hela | 8 | - | - | - |
|  | let-7b | Hela | 32 | √ | √ | - |
| **Georges et al., 2008** | miR-192 | HCT116 | 24 | √ | - | √ |
|  | miR-192 | HCT116 | 10 | √ | - | √ |
|  | miR-215 | HCT116 | 10 | √ | - | - |
|  | miR-215 | HCT116 | 24 | √ | - | √ |
| **Baek et al., 2008** | miR-124 | Hela | 24 | √ | √ | - |
|  | miR-1 | Hela | 24 | √ | √ | - |
|  | miR-181a | Hela | 24 | √ | √ | - |
| **He et al., 2005** | miR-34a | A549 H-1 | 24 | √ | √ | - |
|  | miR-34b | A549 H-1 | 24 | √ | √ | - |
|  | miR-34a | HCT116 | 24 | √ | √ | √ |
|  | miR-34b | HCT116 | 24 | √ | √ | - |
|  | miR-34a | TOV21G H1 | 24 | √ | √ | - |
|  | miR-34b | TOV21G H1 | 24 | √ | √ | - |
|  | miR-34a | DLD | 24 | √ | √ | √ |
|  | miR-34b | DLD | 24 | √ | √ | - |
|  | miR-34a | HeLa | 24 | √ | √ | √ |
|  | miR-34b | HeLa | 24 | √ | √ | - |
|  | miR-34a | A549 p53 | 24 | √ | √ | - |
|  | miR-34b | A549 p53 | 24 | √ | √ | - |
| **Grimson et al., 2007** | miR-7 | Hela | 12 | √ | √ | - |
|  | miR-7 | Hela | 24 | √ | √ | √ |
|  | miR-9 | Hela | 12 | √ | √ | √ |
|  | miR-9 | Hela | 24 | √ | √ | - |
|  | miR-122 | Hela | 12 | √ | √ | - |
|  | miR-122 | Hela | 24 | √ | √ | - |
|  | miR-128 | Hela | 12 | √ | √ | - |
|  | miR-128 | Hela | 24 | √ | √ | √ |
|  | miR-132 | Hela | 12 | √ | √ | √ |
|  | miR-132 | Hela | 24 | √ | √ | - |
|  | miR-133a | Hela | 12 | √ | √ | √ |
|  | miR-133a | Hela | 24 | √ | √ | √ |
|  | miR-142-3p | Hela | 12 | √ | - | √ |
|  | miR-142-3p | Hela | 24 | √ | - | √ |
|  | miR-148b | Hela | 12 | √ | √ | - |
|  | miR-148b | Hela | 24 | √ | √ | √ |
|  | miR-181a | Hela | 12 | √ | √ | - |
|  | miR-181a | Hela | 24 | √ | √ | - |

The transfecting miRNAs have been detected as active in 42 out of 43 miRNA transfection experiments based on Wilcoxon (WR) test. 15 out of 17 transfecting miRNAs in 34 out of 43 miRNA transfection experiments have been identified as active applying the Kolmogorov-Smirnov (KS) test. 13 out of 17 transfecting miRNAs in 18 out of 43 miRNA transfection experiments have been detected applying the hypergeometric (HG) test.

**Table A3** - Prediction of active TFs based on the differential expression of their target genes.

| **Datasets** | **Transfect./over expressed miRNA** | **Cell line** | **Time point** | **Total** | **Fold changed (FC)** | **FC+stat. sig.** | **Statistically (stat.) sig. TFs** | | | | |
| --- | --- | --- | --- | --- | --- | --- | --- | --- | --- | --- | --- |
|  |  |  |  |  |  |  | **WR test** | **KS test** | **Shared KS+WR** | **HG test** | **Shared HG+WR** |
| **Selbach et al., 2008** | miR-155 | HeLa | 8 | 27 | 7 | 0 | 20 | 6 | 6 | 0 | 0 |
|  | miR-155 | Hela | 32 | 30 | 11 | 3 | 16 | 13 | 13 | 6 | 3 |
|  | miR-16 | Hela | 8 | 20 | 9 | 0 | 11 | 2 | 2 | 1 | 0 |
|  | miR-16 | Hela | 32 | 34 | 10 | 0 | 24 | 17 | 17 | 0 | 0 |
|  | let-7b | Hela | 8 | 27 | 5 | 1 | 21 | 9 | 9 | 5 | 3 |
|  | let-7b | Hela | 32 | 25 | 8 | 1 | 16 | 9 | 9 | 5 | 4 |
| **Georges et al., 2008** | miR-192 | HCT116 | 24 | 19 | 2 | 0 | 17 | 12 | 12 | 1 | 0 |
|  | miR-192 | HCT116 | 10 | 25 | 0 | 0 | 25 | 12 | 12 | 0 | 0 |
|  | miR-215 | HCT116 | 10 | 20 | 0 | 0 | 20 | 10 | 10 | 0 | 0 |
|  | miR-215 | HCT116 | 24 | 20 | 1 | 0 | 19 | 10 | 10 | 1 | 0 |
| **Baek et al., 2008** | miR-124 | Hela | 24 | 33 | 4 | 0 | 29 | 31 | 28 | 0 | 0 |
|  | miR-1 | Hela | 24 | 40 | 2 | 0 | 38 | 34 | 34 | 0 | 0 |
|  | miR-181a | Hela | 24 | 22 | 5 | 0 | 17 | 24 | 17 | 0 | 0 |
| **He et al., 2005** | miR-34a | A549 H-1 | 24 | 64 | 0 | 0 | 64 | 62 | 61 | 0 | 0 |
|  | miR-34b | A549 H-1 | 24 | 57 | 0 | 0 | 57 | 56 | 52 | 0 | 0 |
|  | miR-34a | HCT116 | 24 | 65 | 4 | 1 | 60 | 58 | 55 | 0 | 0 |
|  | miR-34b | HCT116 | 24 | 66 | 3 | 0 | 63 | 59 | 58 | 0 | 0 |
|  | miR-34a | TOV21G H1 | 24 | 71 | 0 | 0 | 71 | 59 | 59 | 0 | 0 |
|  | miR-34b | TOV21G H1 | 24 | 64 | 0 | 0 | 64 | 62 | 62 | 0 | 0 |
|  | miR-34a | DLD | 24 | 65 | 1 | 1 | 63 | 60 | 59 | 0 | 0 |
|  | miR-34b | DLD | 24 | 59 | 3 | 1 | 55 | 53 | 51 | 0 | 0 |
|  | miR-34a | HeLa | 24 | 64 | 0 | 0 | 64 | 62 | 60 | 0 | 0 |
|  | miR-34b | HeLa | 24 | 63 | 0 | 0 | 63 | 57 | 56 | 0 | 0 |
|  | miR-34a | A549 p53 | 24 | 61 | 0 | 0 | 61 | 60 | 58 | 0 | 0 |
|  | miR-34b | A549 p53 | 24 | 59 | 0 | 0 | 59 | 60 | 56 | 0 | 0 |
| **Grimson et al., 2007** | miR-7 | Hela | 12 | 9 | 0 | 0 | 9 | 4 | 4 | 0 | 0 |
|  | miR-7 | Hela | 24 | 0 | 0 | 0 | 0 | 0 | 0 | 0 | 0 |
|  | miR-9 | Hela | 12 | 31 | 2 | 0 | 29 | 15 | 15 | 0 | 0 |
|  | miR-9 | Hela | 24 | 9 | 0 | 0 | 9 | 0 | 0 | 0 | 0 |
|  | miR-122 | Hela | 12 | 5 | 3 | 0 | 2 | 1 | 1 | 2 | 1 |
|  | miR-122 | Hela | 24 | 29 | 4 | 1 | 24 | 18 | 17 | 2 | 2 |
|  | miR-128 | Hela | 12 | 4 | 0 | 0 | 4 | 6 | 4 | 0 | 0 |
|  | miR-128 | Hela | 24 | 12 | 1 | 0 | 11 | 8 | 7 | 0 | 0 |
|  | miR-132 | Hela | 12 | 0 | 0 | 0 | 0 | 0 | 0 | 0 | 0 |
|  | miR-132 | Hela | 24 | 0 | 0 | 0 | 0 | 0 | 0 | 0 | 0 |
|  | miR-133a | Hela | 12 | 27 | 5 | 1 | 21 | 14 | 13 | 4 | 2 |
|  | miR-133a | Hela | 24 | 31 | 12 | 2 | 17 | 16 | 16 | 9 | 6 |
|  | miR-142-3p | Hela | 12 | 3 | 0 | 0 | 3 | 2 | 2 | 0 | 0 |
|  | miR-142-3p | Hela | 24 | 2 | 0 | 0 | 2 | 0 | 0 | 0 | 0 |
|  | miR-148b | Hela | 12 | 8 | 1 | 0 | 7 | 6 | 5 | 0 | 0 |
|  | miR-148b | Hela | 24 | 19 | 1 | 0 | 18 | 11 | 11 | 1 | 0 |
|  | miR-181a | Hela | 12 | 0 | 0 | 0 | 0 | 0 | 0 | 0 | 0 |
|  | miR-181a | Hela | 24 | 9 | 0 | 0 | 9 | 5 | 5 | 0 | 0 |
| **Total** |  |  |  | 120 | 52 | 7 | 88 | 73 | 71 | 21 | 12 |

Overall, 120 TFs have been identified by MIRTFnet (applying the Wilcoxon (WR) test) with the used datasets. 73 and 21 TFs have been detected applying the Kolmogorov-Smirnov (KS) and hypergeometric (HG) test in 37 and 11 out of 43 miRNA transfection profiles. The WR, KS and HG test does not find any significant TF in 4, 6 and 32 out of 43 miRNA transfection profiles. In 15 out of 37 miRNA transfection profiles the detected significant TFs overlap between the WR and KS test is 100%. In all of these cases WR test has detected more active TFs than KS test.

## Table A4 - Percentage of differentially expressed genes explained by MIRTFnet

| **Dataset** | **miRNA, Time point** | **Cell Line** | **Diff. exp. genes (down/up)** | **miRNA- downreg. targets** | **miRNA-regulated TF: targets (Sig. TFs)** | **TF-regulated TF: targets (Sign.TFs)** | **Regulated genes (down/up)** | **Percentage combined (down/up) regulated** |
| --- | --- | --- | --- | --- | --- | --- | --- | --- |
|  |  |  |  |  |  |  |  |  |
| **Selbach et al., 2008** | miR-155(8hr) | Hela | 268(189/78) | 57 | 97(6) | 52(5) | 148(109/39) | 55(57/50) |
|  | miR-155(32hr) | Hela | 534(385/148) | 201 | 283(12) | 110(7) | 382(303/79) | 71(78/53) |
|  | miR-16(8hr) | Hela | 360(250/110) | 130 | 108(5) | 57(5) | 211(168/43) | 58(67/39) |
|  | miR-16(32hr) | Hela | 516(269/247) | 174 | 277(15) | 215(10) | 380(235/145) | 73(87/58) |
|  | let-7b(8hr) | Hela | 236(168/67) | 56 | 102(6) | 92(7) | 150(112/38) | 63(66/56) |
|  | let-7b(32hr) | Hela | 259(138/120) | 63 | 117(9) | 66(3) | 154(99/55) | 59(71/45) |
| **Georges et al., 2008** | miR-192(24hr) | HCT116 | 71(40/31) | 33 | 27(6) | 36(6) | 55(38/17) | 77(95/54) |
|  | miR-192(10hr) | HCT116 | 9(5/4) | 5 | 3(3) | 3(2) | 6(5/1) | 66(100/25) |
|  | miR-215(10hr) | HCT116 | 9(5/4) | 5 | 3(2) | 6(3) | 7(5/2) | 77(100/50) |
|  | miR-215(24hr) | HCT116 | 105(49/56) | 45 | 30(5) | 38(4) | 70(47/23) | 66(95/41) |
| **Baek et al., 2008** | miR-124(24hr) | Hela | 324(171/153) | 65 | 164(18) | 120(10) | 203(120/83) | 62(70/54) |
|  | miR-1(24hr) | Hela | 143(26/117) | 8 | 62(15) | 68(13) | 82(17/65) | 57(65/55) |
|  | miR-181a(24hr) | Hela | 399(205/194) | 44 | 121(9) | 158(8) | 200(107/93) | 50(52/47) |
| **He et al., 2005** | miR-34a(24hr) | A549 | 48(19/29) | 12 | 24(28) | 25(24) | 33(15/18) | 68(78/62) |
|  | miR-34b(24hr) | A549 | 27(8/19) | 3 | 8(15) | 10(22) | 12(4/8) | 44(50/42) |
|  | miR-34a(24hr) | HCT116 | 150(98/52) | 75 | 93(27) | 82(24) | 127(92/35) | 84(93/67) |
|  | miR-34b(24hr) | HCT116 | 131(71/60) | 30 | 77(24) | 83(34) | 96(56/40) | 73(78/66) |
|  | miR-34a(24hr) | TOV21G | 11(7/4) | 5 | 5(11) | 5(10) | 9(6/3) | 81(85/75) |
|  | miR-34b(24hr) | TOV21G | 7(6/1) | 3 | 1(1) | 1(0) | 4(4/0) | 57(66/0) |
|  | miR-34a(24hr) | DLD | 126(97/29) | 72 | 84(30) | 69(25) | 108(88/20) | 85(90/68) |
|  | miR-34b(24hr) | DLD | 202(123/79) | 46 | 111(19) | 120(30) | 145(94/51) | 71(76/64) |
|  | miR-34a(24hr) | HeLa | 57(39/18) | 29 | 23(29) | 24(24) | 40(31/9) | 70(79/50) |
|  | miR-34b(24hr) | HeLa | 67(39/28) | 19 | 32(22) | 37(32) | 42(30/12) | 62(76/42) |
|  | miR-34a(24hr) | A549 | 59(27/32) | 19 | 37(30) | 35(23) | 45(24/21) | 76(88/65) |
|  | miR-34b(24hr) | A549 | 40(18/22) | 4 | 15(19) | 23(27) | 26(10/16) | 65(55/72) |
| **Grimson et a., 2007** | miR-7(12hr) | Hela | 68(39/29) | 27 | 5(1) | 14(1) | 37(32/5) | 54(82/17) |
|  | miR-7(24hr) | Hela | 34(28/6) | 19 | 0(0) | 0(0) | 19(19/0) | 55(67/0) |
|  | miR-9(12hr) | Hela | 110(45/65) | 28 | 42(9) | 23(6) | 63(35/28) | 57(77/43) |
|  | miR-9(24hr) | Hela | 14(13/1) | 6 | 0(0) | 0(0) | 6(6/0) | 42(46/0) |
|  | miR-122(12hr) | Hela | 337(181/156) | 56 | 0(0) | 0(0) | 56(54/2) | 16(29/1) |
|  | miR-122(24hr) | Hela | 654(360/294) | 165 | 295(8) | 174(7) | 404(255/149) | 61(70/50) |
|  | miR-128(12hr) | Hela | 51(44/7) | 19 | 4(2) | 2(1) | 23(22/1) | 45(50/14) |
|  | miR-128(24hr) | Hela | 88(56/32) | 44 | 27(5) | 9(1) | 52(47/5) | 59(83/15) |
|  | miR-132(12hr) | Hela | 104(78/26) | 51 | 0(0) | 0(0) | 51(51/0) | 49(65/0) |
|  | miR-132(24hr) | Hela | 52(28/24) | 12 | 0(0) | 0(0) | 12(12/0) | 23(42/0) |
|  | miR-133a(12hr) | Hela | 77(31/46) | 29 | 29(9) | 29(6) | 54(28/26) | 70(90/56) |
|  | miR-133a(24hr) | Hela | 267(107/160) | 82 | 120(10) | 128(12) | 186(94/92) | 69(87/57) |
|  | miR-142-3p(12hr) | Hela | 126(92/34) | 41 | 8(1) | 0(0) | 44(44/0) | 34(47/0) |
|  | miR-142-3p(24hr) | Hela | 54(25/29) | 17 | 0(0) | 0(0) | 17(17/0) | 31(68/0) |
|  | miR-148b(12hr) | Hela | 32(16/16) | 5 | 5(3) | 3(1) | 11(7/4) | 34(43/25) |
|  | miR-148b(24hr) | Hela | 257(104/153) | 66 | 55(2) | 67(6) | 128(78/50) | 49(75/32) |
|  | miR-181a(12hr) | Hela | 26(22/4) | 8 | 0(0) | 0(0) | 8(8/0) | 30(36/0) |
|  | miR-181a(24hr) | Hela | 54(29/25) | 14 | 9(2) | 8(2) | 23(19/4) | 42(65/16) |

In case of the miR-155 transfection at 8hr, 148/268=55% of the differentially expressed genes can be explained by the union of the 67 miR-155 target genes, the 97 targets of the 6 TFs directly targeted by miR-155 and the 52 targets of the 5 TFs indirectly affected by miR-155. Indirectly affected TFs are connected to miR-155 by miRNA-(kinase-)(TF-…-)TF chains. The 6+5 TFs were identified as active TFs by MIRTFnet (applying the Wilcoxon (WR) and Kolmogorov-Smirnov (KS) tests). We regard genes as differentially expressed if they exhibit a fold change of at least 2 or less than 0.5.

**Table A5 - Area under the ROC (AUROC) analysis for the Wilcoxon (WR), Kolmogorov-Smirnov (KS), and hypergeometric (HG)** test using 25 miRNA transfections.

| **miRNA-gene target source** | **Tested secondary miRNAs** | **AUROC** | | |
| --- | --- | --- | --- | --- |
|  |  | **WR test** | **KS test** | **HG test** |
| **Databases (DB)** | 314 | 0.73 | 0.68 | 0.62 |
| **DB+ PICTAR+TargetScan (P1)** | 404 | 0.76 | 0.75 | 0.71 |
| **P1+PITA(threshold of -20)** | 775 | 0.81 | 0.79 | 0.62 |
| **P1+PITA(threshold of -11)** | 780 | 0.67 | 0.65 | 0.57 |
| **P1+PITA(threshold of -6)** | 780 | 0.67 | 0.66 | 0.57 |

The performance of each method has been accessed by means of area under the ROC curve. The Wilcoxon (WR), Kolmogorov-Smirnov (KS) and hypergeometric (HG) test has been applied to determine the significance of each miRNA (i.e., primary/transfecting and secondary miRNA) using the databases and prediction tools miRNA-gene target associations in all 25 miRNA transfections. For AUROC the primary or the transfecting miRNA in each transfecting experiment is considered as positive example (which are 25 in total) and the rest of the miRNAs are considered as negative examples. Using the databases miRNA-gene pairs the WR, KS and HG test achieve the AUROC of 0.73, 0.68 and 0.62. Combining the databases and prediction tools (e.g., PICTAR and TargetScan) miRNA-gene target associations improves the AUROC curve. In case of PITA predictions (filtered at a threshold of -11 and -6) decreases the AUROC. While using the PITA predictions at stringent threshold improves the AUROC. Overall, the WR test achieves the best AUROC in comparison to KS and HG test.

**Table A6 - Area under the ROC (AUROC) analysis for the Wilcoxon (WR), Kolmogorov-Smirnov (KS), and hypergeometric (HG) test using 43 miRNA transfections.**

| **miRNA-gene target source** | **Tested secondary miRNAs** | **AUROC** | | |
| --- | --- | --- | --- | --- |
|  |  | **WR test** | **KS test** | **HG test** |
| **Databases (DB)** | 314 | 0.63 | 0.59 | 0.58 |
| **DB+ PICTAR+TargetScan (P1)** | 404 | 0.88 | 0.87 | 0.70 |
| **P1+PITA(threshold of -20)** | 775 | 0.9 | 0.86 | 0.65 |
| **P1+PITA(threshold of -11)** | 780 | 0.71 | 0.69 | 0.58 |
| **P1+PITA(threshold of -6)** | 780 | 0.68 | 0.67 | 0.60 |

The performance of each method has been accessed by means of area under the ROC curve. The Wilcoxon (WR), Kolmogorov-Smirnov (KS) and hypergeometric (HG) test has been applied to determine the significance of each miRNA (i.e., primary/transfecting and secondary miRNA) using the databases and prediction tools miRNA-gene target associations in all 43 miRNA transfections. For AUROC the primary or the transfecting miRNA in each transfecting experiment is considered as positive example (which are 43 in total) and the rest of the miRNAs are considered as negative examples. Using the databases miRNA-gene pairs the WR, KS and HG test achieve the AUROC of 0.63, 0.59 and 0.58. Combining the databases and prediction tools (e.g., PICTAR and TargetScan) miRNA-gene target associations improves the AUROC curve. In case of PITA predictions (filtered at a threshold of -11 and -6), the AUROC improves in comparison to databases but decreases in comparison to combine PICTAR, TargetScan and databases miRNA target associations. Combining the PITA predictions at stringent threshold (of -20) improves the AUROC of WR test in comparison to KS and HG test.

## Models connecting the transfecting miRNA to active TFs

Transfection experiment specific models connect the transfecting miRNA via relationships (derived from databases or predictions) to transcription factors (TFs) detected as active by MIRTFnet (applying the Wilcoxon (WR) and Kolmogorov-Smirnov (KS) test statistics) directly or via kinases. Each transfection model contains miRNA-TF, miRNA-kinase, kinase-TF and TF-TF associations. The number interactions contained in the experiment specific models are shown in Table A4 (columns 6 and 7). Files including the experiment specific models are provided upon request, due to the large number of model files (~100).

## Table A7 - miRNA-(kinase)-TF regulatory models identified based on MIRTFnet

| **Columns** | **Description** |
| --- | --- |
| **InteractorA** | Either miRNA, kinase, or TF NCBI official gene name |
| **InteractorB** | InteractorA-regulated either kinase (in case of miRNA) or TF (in case of miRNA, kinase or TF) NCBI official gene name |
| **InteractorA gene-ID** | NCBI Entrez gene ID for InteractorA, i.e. the regulator |
| **InteractorB gene-ID** | NCBI Entrez gene ID for InteractorB, i.e. the regulated target |
| **Type** | Specifies the kind of relationship between InteractorA and InteractorB (i.e., miRNA-kinase, kinase-TF and TF-TF associations) |
| **Experiment-ID** | Experiment specific identifier. Please see the Table A1 for more detail |
| **Dataset-ID** | Dataset (i.e. array-) specific identifier. Please see the Table A1 for more detail |
| **Time point** | miRNA-transfection experiment time point (hr) i.e., 8, 10, 24 and 32 |
| **Databases-resource** | Source(s) from which the InteractorA-InteractorB association has been extracted |

## miRNA-target associations

File S2 contains the transfecting miRNA target genes including TFs, kinases and other differentially regulated miRNA target genes. We regard target genes as differentially expressed if they exhibit a fold change of at least 2 or less than 0.5. Table A8 describes the information contained in File S2.

## Table A8 - miRNA-regulated target genes including TF and kinases associations

| **Columns** | **Description** |
| --- | --- |
| **MiRNA** | Name of the transfecting miRNAs |
| **Target** | miRNA-regulated target: NCBI Entrez gene name |
| **Gene-ID** | miRNA-regulated target: NCBI Entrez gene ID |
| **FoldChange** | Log_2_ fold-changes (l_2_fc) value for the target gene |
| **Databases-resource** | Resource(s) of miRNA-target gene association has been extracted (e.g., miRSel, TarBase, PITA) |
| **Experiment-ID** | Experiment specific identifier. Please see the Table A1 for more detail |
| **Dataset-ID** | Dataset (i.e. array-) specific identifier. Please see the Table A1 for more detail |
| **Pvalue** | P-value for the transfecting miRNA applying the Wilcoxon (WR) test |
| **PvalueKStest** | P-value for the transfecting miRNA applying the Kolmogorov-Smirnov (KS) test |
| **Cell Line** | Cell line of miRNA-transfection study |
| **Time point** | miRNA-transfection experiment time point (hr) i.e., 8, 10, 24 and 32 |
| **Type** | (0: highly differentially expressed miRNA-regulated target gene, 1: significant TF (statistically or fold change based) regulated by miRNA, 2: highly differentially expressed miRNA-regulated kinase, 3- miRNA-regulated kinase associated with significant TF(s) |

## Kinase-TF relationships

File S3 lists the kinase-TF associations necessary to link active TF to the transfecting miRNA in each miRNA-transfection experiment. Note that kinases are included as connectors between miRNAs and significant TFs in the models although the activity of kinases has not been determined in the examined studies. Table A9 describes the information contained in File S3.

## Table A9 - kinase-TF associations

| **Columns** | **Description** |
| --- | --- |
| **Kinase** | Regulator (i.e. kinase) NCBI Entrez gene name |
| **Kinase-Gene-ID** | Kinase NCBI Entrez gene ID |
| **Target** | Kinase-targeted TF NCBI Entrez gene name |
| **Target-Gene-ID** | Kinase-targeted TF Entrez gene ID |
| **Kinase-FC** | Log_2_ fold-changes (l_2_fc) value for the kinase gene |
| **Target-FC** | Log_2_ fold-changes (l_2_fc) value for the kinase targeted TF gene |
| **Experiment-ID** | Experiment specific identifier. Please see the Table A1 for more detail. |
| **Dataset-ID** | Dataset (i.e. array-) specific identifier. Please see the Table A1 for more detail. |
| **Time point** | miRNA-transfection experiment time point (hr) i.e., 8, 10, 24 and 32 |

## Significant TF

Overall, 120 TFs have been identified as active by MIRTFnet in the examined datasets. File S4 contains the TFs identified as active (either based on Wilcoxon and Kolmogorov-Smirnov test or based on the fold change criterion) in each miRNA transfection experiment. Table A10 describes the information contained in File S4.

## Table A10 – TFs identified as active by MIRTFnet in the examined datasets

| **Columns** | **Description** |
| --- | --- |
| **TFname** | Gene name of the TF detected as active by MIRTFnet |
| **TF-Gene-ID** | Active TF NCBI Entrez gene ID |
| **Pvalue-BH** | P-value for statistically active TF applying the Wilcoxon (WR) test(multiple testing correction according to the Benjamini-Hochberg method) |
| **Pvalue-KS-BH** | P-value for statistically active TF applying the Kolmogorov-Smirnov (KS) test (multiple testing correction according to the Benjamini-Hochberg method) |
| **FoldChange** | Lold-changes (l_2_fc) value for the active TF |
| **TargetedBymiRNAorTF** | Gene IDs of the regulator(s) (either miRNA(s) or TF(s)) of the given active TF |
| **Cell Line** | Cell line of miRNA-transfection study |
| **Experiment-ID** | Experiment specific identifier. Please see the Table A1 for more detail. |
| **Dataset-ID** | Dataset (i.e. array-) specific identifier. Please see the Table A1 for more detail |
| **Time point** | miRNA-transfection experiment time point (hr) i.e., 8, 10, 24 and 32 |
| **TransfectedmiRNArelated** | Statistically active TF either directly/indirectly connected to the primary miRNA (1) or not (0). |

## TF-target relationships

The identified active TFs and their target genes (in addition to the direct miRNA target genes) provide a potential explanation for the majority of the observed differential expression in the 43 examined miRNA transfection experiments. We regard target genes as differentially expressed if they exhibit a fold change of at least 2 or less than 0.5. File S5 contains the active TF regulated target genes information, see Table A11.

## Table A11 - TF-regulated target genes relationships

| **Columns** | **Description** |
| --- | --- |
| **TFname** | Active TF gene name |
| **Target** | TF-regulated target NCBI Entrez gene name |
| **TF-Gene-ID** | Active TF NCBI Entrez gene name |
| **Target-Gene-ID** | TF-regulated target Entrez gene ID |
| **Target-FC** | Log_2_ fold-changes (l_2_fc) value for the TF-target gene |
| **Databases-resource** | Resource(s) where the TF-target gene association has been extracted from (e.g., UCSC, JASPAR and TRANSFAC) |
| **Experiment-ID** | Experiment specific identifier. Please see the Table A1 for more detail. |
| **Dataset-ID** | Dataset (i.e. array-) specific identifier. Please see the Table A1 for more detail |
| **Time point** | miRNA-transfection experiment time point (hr) i.e., 8, 10, 24 and 32 |
| **Cell Line** | Cell line of miRNA-transfection study |

## miRNA-TF core regulatory network

We constructed an intersection model from the TFs contained in at least 7 of the 19 (compare Table A1, column ‘Counted’) individual miRNA transfection models. Because of their special importance, we also added p53, NFKB1 and PAX5 although they are only found active in 4, 6 and 6 of the models. The resulting intersection model contains 21 TFs. Figure A1 depicts putative relationships as derived from the STRING database [6] for 19 out of 21 identified significant TFs.


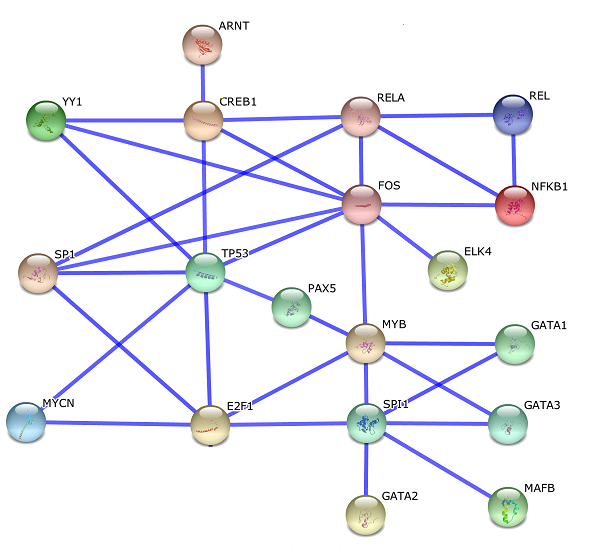


**Figure A1 -** TF mediators of miRNA-triggered regulation active in at least 7 out of 19 miRNA-transfection experiments. The edges show putative associations between TFs derived from the STRING database (http://string-db.org/) at a significance threshold of 0.9.

## References

1. Selbach M, Schwanhäusser B, Thierfelder N, Fang Z, Khanin R, Rajewsky N: **Widespread changes in protein synthesis induced by microRNAs.** *Nature* 2008, **455:**58-63.
2. He L, He X, Lim LP, de Stanchina E, Xuan Z, Liang Y, Xue W, Zender L, Magnus J, Ridzon D, Jackson AL, Linsley PS, Chen C, Lowe SW, Cleary MA, Hannon GJ: **A microRNA component of the p53 tumour suppressor network.** *Nature* 2007, **447:**1130-1134.
3. Georges SA, Biery MC, Kim SY, Schelter JM, Guo J, Chang AN, Jackson AL, Carleton MO, Linsley PS, Cleary MA, Chau BN: **Coordinated regulation of cell cycle transcripts by p53-Inducible microRNAs, miR-192 and miR-215.** *Cancer Res* 2008, **15:**10105-12.
4. Baek D, Villén J, Shin C, Camargo FD, Gygi SP, Bartel DP: **The impact of microRNAs on protein output.** *Nature* 2008, **455:**64-71.
5. Grimson A, Farh KK, Johnston WK, Garrett-Engele P, Lim LP, Bartel DP: **MicroRNA targeting specificity in mammals: determinants beyond seed pairing**. *Mol Cell* 2007, 27:91-105.
6. Jensen LJ, Kuhn M, Stark M, Chaffron S, Creevey C, Muller J, Doerks T, Julien P, Roth A, Simonovic M, Bork P, von Mering C: **STRING 8--a global view on proteins and their functional interactions in 630 organisms.** *Nucleic Acids Res* 2009, **37:**D412-6.
